# Crosstalk between Blood Vessels and Glia during the Central Nervous System Development

**DOI:** 10.3390/life12111761

**Published:** 2022-11-01

**Authors:** Hidenori Tabata

**Affiliations:** Department of Molecular Neurobiology, Institute for Developmental Research, Aichi Developmental Disability Center, 713-8 Kamiya, Kasugai 480-0392, Japan; tabata@inst-hsc.jp; Tel.: +81-568-88-0811 (ext. 7513)

**Keywords:** astrocyte, oligodendrocyte, blood–brain barrier

## Abstract

The formation of proper blood vessel patterns in the central nervous system (CNS) is crucial to deliver oxygen and nutrient to neurons efficiently. At the same time, neurons must be isolated from the outer blood circulation by a specialized structure, the blood–brain barrier (BBB), to maintain the microenvironment of brain parenchyma for the survival of neurons and proper synaptic transmission. To develop this highly organized structure, glial cells, a major component of the brain, have been reported to play essential roles. In this review, the crosstalk between the macroglia, including astrocytes and oligodendrocytes, and endothelial cells during the development of CNS will be discussed. First, the known roles of astrocytes in neuro-vascular unit and its development, and then, the requirements of astrocytes for BBB development and maintenance are shown. Then, various genetic and cellular studies revealing the roles of astrocytes in the growth of blood vessels by providing a scaffold, including laminins and fibronectin, as well as by secreting trophic factors, including vascular endothelial growth factor (VEGF) and transforming growth factor-β (TGF-β) are introduced. Finally, the interactions between oligodendrocyte progenitors and blood vessels are overviewed. Although these studies revealed the necessity for proper communication between glia and endothelial cells for CNS development, our knowledge about the detailed cellular and molecular mechanisms for them is still limited. The questions to be clarified in the future are also discussed.

## 1. Introduction

During the development of the central nervous system (CNS), neural stem cells produce neurons and macroglia, including astrocytes and oligodendrocytes [1,2,3]. These brain-intrinsic cells occupy most of the space within the brain parenchyma. Additionally, the brain also adopts extrinsic cells in the early stages of development, namely endothelial cells and microglia [4,5]. These brain-intrinsic and -extrinsic cells do not develop independently but interact with each other intimately to construct the brain. In this review, the interplays between macroglia and endothelial cells during CNS development are described (for microglia, see another chapter on this Special Issue).

The development of glia remains largely unknown [6]. They are produced in the ventricular zone, and migrate into the final destination like neurons do. However, even fundamental information regarding their development, such as their migration patterns, the scaffold they relying on, and determination mechanisms of their destination, is still obscure. Nevertheless, the recent advantages of the lineage-tracing technique revealed that astrocyte positioning in the cortical gray matter highly coincided with synaptogenesis and blood vessel remodeling, suggesting their involvement in these processes [7]. Blood vessels are windows to the outer environment of the brain parenchyma. Detrimental physical changes or chemicals can affect glia-blood vessel interactions, especially during the developmental period, which in turn can lead to long-lasting deficits, highlighting the importance of the studies to reveal the basic mechanisms of them and their perturbation factors.

In this review, our knowledge about glia-blood vessel interactions and the underlying molecular mechanisms will be discussed. First, the physiological functions of the neurovascular unit, its development, and the roles of glia (astrocytes) in it is introduced. Then, the interplay between glia and blood vessels during the development of the retina are shown, in which the cellular and molecular mechanisms have been well investigated. Additionally, their interaction in the developing cerebral cortex is overviewed. Finally, the interaction between oligodendrocytes and blood vessels is discussed.

## 2. Astrocytes Form Neurovascular Units

**Figure 1:**
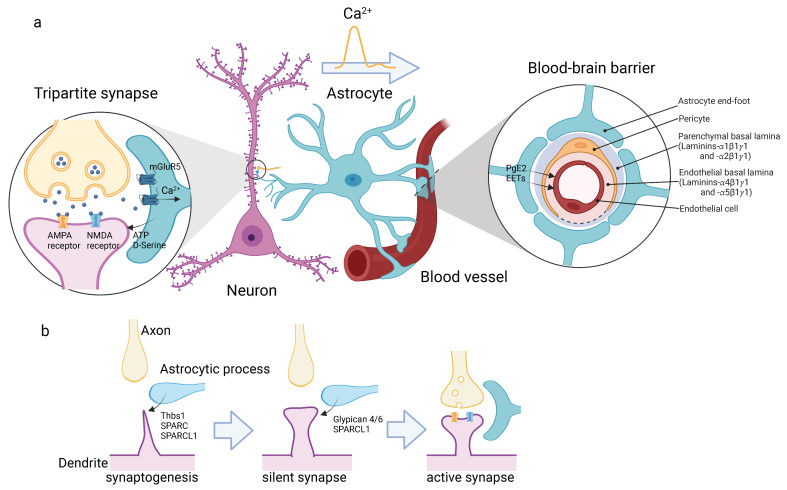
Astrocytes form neurovascular units. (**a**) Astrocytes connect neurons and blood vessels directly. In the cortical gray matter, astrocytes extend highly branched thin processes, whose tips enclose the pre- and post-synapses of neurons. This structure is called “tripartite synapse” (**left**). Astrocytes sense neurotransmitters via glutamate receptors, such as mGluR5, which propagate Ca^2+^ transients in astrocytes. Astrocytes release gliotransmitter, ATP and D-Serine, in response to the Ca^2+^ transient to regulate the synaptic activity. Astrocytes also extend their end-feet and wrap around blood vessels to form the outer most structure of the BBB (**right**). The schematic drawing shows cross section of BBB. The compartments of basement membrane are shown. Ca^2+^ waves travel across the cell body and reach the end-feet, and trigger the release of prostaglandin E2 (PgE2) and epoxyeicosatrienoic acids (EETs). (**b**) Development of tripartite synapse. Astrocytic process releases SPARC and SPARCL1 to induce dendritic spine formation (synaptogenesis, **left**), and then Glypican 4/6 and SPARCL1 to maturate synapses from inactive state (silent synapse, **middle**) to active state (**right**). Astrocytic processes tightly wrap the synapse to form mature tripartite synapse. The illustrations are created with BioRender.com.

In the cortical gray matter, astrocytes extend highly branched thin processes that form mutually exclusive territories. This type is called the protoplasmic astrocyte. The tips of their fine processes enclose the pre- and post-synapses of neurons, and this structure is called the “tripartite synapse” [8] (Figure 1a). Through this structure, astrocytes rapidly uptake neurotransmitters from the synaptic cleft through their transporters to prevent prolonged and/or excessive firing of the post-synaptic neurons. Astrocytes also sense neurotransmitters that are released from pre-synapses via their glutamate receptors, such as mGluR5, which in turn propagate Ca^2+^ transients in astrocytes. This leads to the release of “gliotransmitter”, such as ATP and D-serine [9] back into the synaptic cleft. Thus, astrocytes modulate the efficiency of synaptic transmission. Additionally, astrocytes also extend their end-feet and wrap around blood vessels to form the outer most structure of the BBB [10]. In this way, astrocytes directly connect neurons and blood vessels to form functional neurovascular units. For example, Ca^2+^ elevation in astrocytes triggered by synaptic activity leads to the release of prostaglandin E2 [11,12] or epoxyeicosatrienoic acids [13], both of which are products of the arachidonic acid cascade, which in turn enhances the dilation of the smooth muscles of the arteriole. These consecutive reactions enable the brain to increase its blood flow in response to neuronal activity. To develop this system, astrocytes have to interact with neural synapses and blood vessels. Astrocyte progenitors migrate into the cortical gray matter by the seventh postnatal day (P7). They then begin to arborize highly branched processes. Simultaneously, neurons in the gray matter begin to arborize their dendrites. Astrocytes address dendrites through their perisynaptic processes and secrete several factors, such as thrombospondin-1 (Thbs1) [14], SPARC-like 1 (SPARCL1), and SPARC [15], to initiate the synaptogenesis (Figure 1b). This newly formed synapse is not active (silent synapse). Subsequently, astrocytes secrete glypicans 4 and 6 [16,17], as well as SPARCL1 [18] to turn the silent synapses into active form [18]. During this maturation process, astrocytic end-feet tightly associate with synapses and form tripartite synapses [19,20].

## 3. Astrocytes Regulate BBB Formation and Maintenance

Neurons in the brain parenchyma are highly isolated from the outer blood flow by the low-permeability walls of the blood vessels. During development, brain endothelial cells begin to express tight junction-associated proteins, such as claudin5, occludin, and ZO1, at the early embryonic stages from E10 in mice in response to Wnt-7a and -7b derived from the neuroepithelium [21,22]. They then acquire barrier function by E15.5 due to the maturation of their tight junctions [23]. Pericytes, one of the cellular components of BBB, are derived from neural crest and enter the brain as early as embryonic day 10 (E10) along with endothelial cells, and associate with blood vessels from the early brain development [24,25]. Mature BBB structures are formed by attachment of astrocytic end-feet to cover the blood vessels together with pericytes. In this structure, two distinct extracellular matrix (ECM)-rich basal laminae are formed, which can be recognized by component of laminin. Laminins are one of the major ECM proteins composed of three subunits: α, β, and γ chains [26]. Astrocytes deposit the parenchymal basal lamina containing laminins α1β1γ1 and α2β1γ1 between pericytes and the astrocyte end-feet [27], whereas the endothelial cells form the endothelial basal lamina containing laminins-α4β1γ1 and -α5β1γ1 at the perivascular side of them (Figure 1a). Although astrocytes can increase the expression level of claudin5 and occludin in endothelial cells by transforming growth factor-β (TGF-β) signaling in vitro [28], their involvement in the initial acquisition of barrier function is not presumable as it takes place ahead of astrogliogenesis. Nevertheless, astrocytes play a role in stabilizing the structure and function of the mature BBB. In fact, in astrocyte-specific conditional laminin 𝛾1 subunit KO mice (Nestin-Cre;Lamc1-flox), pericyte differentiation was altered (upregulation of Acta2, a marker for smooth muscle), and the vasculature became leaky [29]. Similar phenotypes were observed in laminin α2 KO mice, in which altered endothelial basal lamina, reduced pericyte coverage, and tight junction abnormalities were observed [30]. Mlc1 is expressed specifically in vessel-associated astrocytes but not in those free from blood vessels [31]. By taking advantage of this, Morales et al. specifically eliminated vessel-associated astrocytes [32]. They generated Mlc1-T2A-CreERT2 mice, in which tamoxifen-inducible Cre was expressed in Mlc1 expressing cells. They then crossed them with R26-DTR mice, which expressed the diphtheria toxin receptor in response to the Cre recombinase activity. As a result, the localization of endothelial junction proteins, such as claudin5, was disorganized, and the permeability of blood vessels was increased. These experiments indicate the importance of astrocytes in the maintenance of the functional BBB. The function of Mlc1 is of particular interest. Mlc1 is responsible for megalencephalic leukoencephalopathy with subcortical cysts (MLC). Approximately 75% of patients with MLC have mutations in the MLC1 gene, and in approximately 20% of cases, GlialCAM (also called HEPACAM) is mutated. Patients develop macrocephaly during infancy, which presents as progressive neurological deterioration [31]. These two proteins are colocalized on the plasma membrane of astrocytes facing the perivascular basal lamina of the BBB [31,33], and together with chloride channels such as CLCN2 [34], supposedly dispose potassium ions outside the brain to regulate extracellular ion and water homeostasis in the brain [35]. In this context, the end-feet of astrocytes act as ion/water channels and not as part of the barrier component. We should consider the broader functions of astrocytes in BBB other than the barrier function.

## 4. The Interaction between Astrocytes and Blood Vessels during Retinal Development

**Figure 2:**
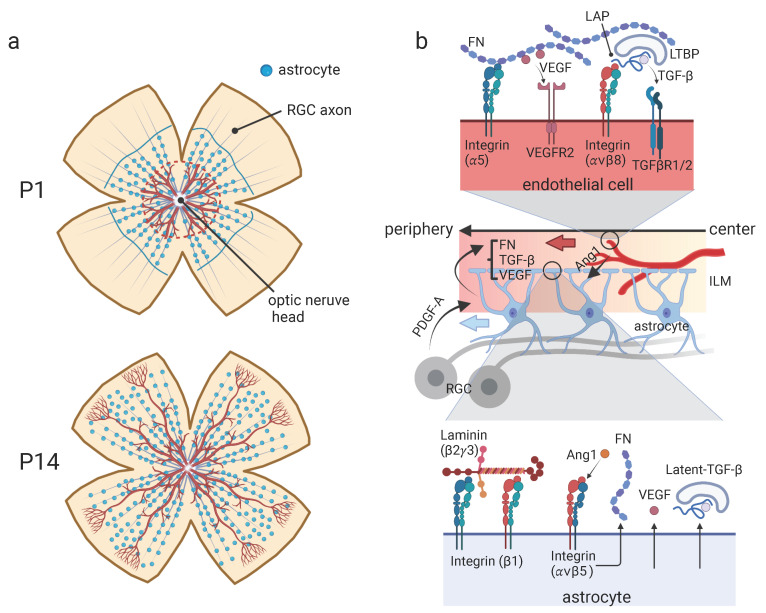
The interaction between astrocytes and blood vessels during retinal development. (**a**) Schematic illustrating of whole retina flat mounts at P1 (**upper**) and P14 (**lower**) in mice. During retinal development, astrocytes migrate into the retina from the optic nerve head and spread peripherally along the axons of retinal ganglion cells (RGCs), and then blood vessels grow on the astrocytes. The blue and red lines in upper panel indicate the area covered by astrocytes and blood vessels, respectively. (**b**) Crosstalk between RGCs, astrocytes, and blood vessels during the retinal development. RGCs express PDGF-A to attract astrocytes. Astrocytes secrete FN, TGF-β and VEGF to regulate angiogenesis, while endothelial cells secrete Ang1 (**middle**), which upregulates expression of FN from astrocytes (**bottom**). The thick blue and red arrows indicate the moving and growing directions of astrocytes and blood vessels, respectively. The receptors and ligands involved in migration/extension of astrocytes and blood vessels are shown in (**upper**) and (**lower**) panels. The illustrations are created with BioRender.com.

Glia-blood vessel interactions have been extensively studied in developing retinas. During retinal development, astrocytes and blood vessels (endothelial cells) migrate into the retina from the optic nerve head, which is the outlet of the optic nerve, and spread peripherally to cover the entire retina (Figure 2a). During this process, astrocytes enter the retina and spread first. This is then followed by blood vessels. This unique feature provides an excellent model for investigating the interaction between astrocytes and blood vessels during pattern formation.

Retinal astrocytes require the axons of retinal ganglion cells (RGCs) for migration. This was proven by a study showing that the lack of RGCs in Math5 knockout mice (KO) resulted in severe astrocyte migration defects [36]. On the other hand, the extension of blood vessels from the center to periphery in developing retina requires astrocytes. The elimination of retinal astrocytes using glial fibrillary acidic protein (GFAP) promoter-Cre (GFAP-Cre) mice caring conditional diphtheria toxin receptor (cDTR) allele resulted in impaired angiogenesis [36]. These observations indicate the sequential patterning mechanism from RGC-axons to astrocytes, and to blood vessels. Two principal mechanisms are assumed to involve in these interactions: trophic factors and scaffolds. During the retinal development, migrating astrocytes highly associate with the axons of RGCs [36]. This is accomplished by Platelet-Derived Growth Factor (PDGF) signaling. Retinal astrocytes express PDGF receptor α (PEGFRα). They are attracted by its ligand, PDGF-A, derived from retinal ganglion cells (RGCs) [37] (Figure 2b). Retinal astrocytes are also relying on scaffolds for migration. They migrate just beneath the inner limiting membrane (ILM). This structure facing the vitreous body forms the retinal basement membrane containing laminin β2 and 𝛾3 chains [38], which are provided by neuroepithelial cells and Müller glia [38]. KO mice of the genes for these subunits (Lamb2 and Lamc3) showed delayed migration of astrocytes and disturbed vascular integrity [39]. The main laminin receptors are integrins, which are composed of α and β subunits [40]. In retinal astrocytes, integrin β1, one of the β subunits of integrins, is strongly expressed and plays central role for migration on ECM. It has been shown that integrin β1 gene (Itgb1)-null astrocytes have defects in polarization, process extension, and migration in a wound healing assay in vitro [41]. Functional blocking of integrin β1 by specific antibodies in explant culture of retinas showed delayed migration of astrocytes, indicating that binding of integrin β1 to laminins in ILM containing β2 and γ3 subunits is crucial for proper migration of retinal astrocytes. Regarding the interactions between astrocytes and blood vessels, astrocytes were shown to provide both scaffolds and trophic factors to endothelial cells. Stenzel et al. reported that astrocytic fibronectin (FN), another ECM protein, and vascular endothelial growth factor (VEGF), a strong trophic factor specifically expressed in astrocytes [42], were important for retinal angiogenesis [43]. They generated astrocyte-specific FN KO mice (GFAP-Cre;FN-flox) and observed reduced migration of blood vessels in their retina without affecting the distribution of astrocytes. The receptors for FN were integrins α5β1, αvβ3, αvβ5, αvβ6, and αvβ8 [40]. Stenzel et al. assessed the retinal blood vessels in endothelial cell-specific Integrin α5 KO mice (Tie2-Cre;Itgba5-flox) and reported the defects in the filopodia formation of endothelial cells, although the migration was not significantly affected, as observed in FN KO mice. Interestingly, in GFAP-Cre;FN-flox mice, VEGF signaling through its receptor, VEGFR2, was disrupted, indicating that VEGF signaling was FN-dependent. These observations suggest that FN acts as a scaffold of the filopodia of endothelial cells, which in part relies on integrin α5, and is also involved in VEGF signaling. The involvement of FN in VEGF signaling has been previously investigated. VEGF binds to a part of the heparin II domain of the FN (type III repeats 13 to 14) [44], and, by adding free peptide of this region to endothelial cells plated on FN-coated dishes, the enhanced migration of endothelial cells by VEGF is significantly reduced, indicating that FN provides a niche for VEGF signaling instead of simply acting as a scaffold. Lee et al. demonstrated the involvement of another trophic factor, Angiopoietin-1 (Ang1), in this signaling system [45]. Ang1 secreted from tip cells of blood vessels acts on growing blood vessels themself through a specific receptor, Tie2, to promote vessel remodeling, maturation, and stabilization [45,46]. On the other hand, Ang1 binds to integrin αvβ5 on astrocytes. Ang1 transcriptionally enhances the expression of FN in astrocytes [45]. These finding indicates that there is indeed a reciprocal feedback loop between Ang1-expressing endothelial cells and FN- and VEGF-expressing astrocytes. TGF-β is another strong trophic factor for blood vessel growth and sprouting [47]. It is secreted into the extracellular space as a latent form. It must be activated before binding to the Tgfbr2 receptor, which is expressed on endothelial cells. Hirota et al. demonstrated that this activation is induced by astrocytes [48]. Latent-TGF-β is a protein complex composed of TGF-β, latent TGF-β binding protein (LTBP), and latency-associated peptide (LAP) [49]. LTBP anchors this protein complex into ECM proteins, such as FN and Fibrillin [50]. Retinal astrocytes express integrin αvβ8, which binds to LAP on its arginine-glycine-aspartic acid (RGD) amino acid sequence. This enables membrane type 1-matrix metalloproteases (MT1-MMP, or MMP14) to access LAP and cleave it, thereby releasing active TGF-β [51]. Hirota et al. used Nestin-Cre mice for the selective ablation of Integrin αv (Itgav) and β8 (Itgb8) in astrocytes, which lowered the density of blood vessels and induced intraretinal hemorrhage [48]. However, another group showed that Itgb8 was expressed in Müller glia [52]. Taken together, retinal astrocytes (or Müller glia) locally activate TGF-β to regulate blood vessel formation in the retina. This complex activation system may be useful for the directional or organized activation of the receptor.

## 5. Interaction between Astrocytes and Blood Vessels during Cortical Development

**Figure 3:**
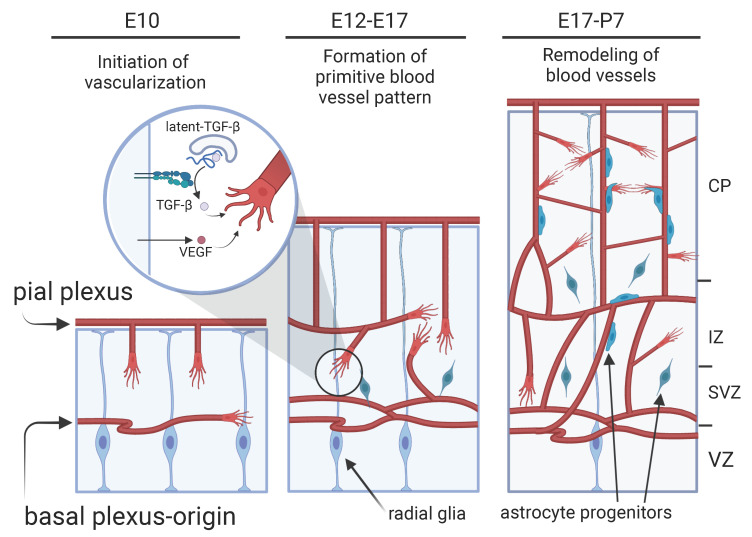
Blood vessel formation and its relationship with radial glia and astrocytes in the cerebral cortex. The blood vessels enter the brain parenchyma from pial and basal plexuses at E10 (**left**). Primitive blood vessel pattern is formed during the embryonic period (E12-17, **middle**). E15 onward, astrocytogenesis starts. From the late prenatal stages to the first postnatal week (E17-P7, **right**), the blood vessels undergoes remodeling. During this period, astrocytes are actively generated and migrate toward brain surface, there they associate with blood vessels, while the radial glia is decreased. The molecules involved in the angiogenesis are shown in a circle window. The initial steps of vascularization are conducted by radial glia. On the other hand, there is a possibility that the remodeling of blood vessels in the later stages is accomplished by astrocytes. The illustrations are created with BioRender.com.

The development of astrocytes in the cerebral cortex remains obscure. After neurogenesis ends around E15 in mice, radial glia in the cortical ventricular zone produce astrocyte progenitors, which migrate toward the cortical gray matter or white matter and differentiate into protoplasmic astrocytes and fibrous astrocytes, respectively, by an unknown mechanism [7,53]. The vigorous migration of astrocyte progenitors from the ventricular or subventricular zone ceases by around P7. After that, they subsequently undergo local proliferation [7,54]. During this early postnatal stage (second and third postnatal weeks in mice), astrocytes intimately interact with neurons and blood vessels to form neuro-vascular units. At this stage, cortical neurons begin to develop their dendrites and dendritic spines (post-synaptic structure), and astrocytes actively participate in this process through various secreted factors, as shown above [55] (Figure 1b). As for vascularization, unlike the retina, the initial step proceeds independently of astrocytes because this process starts as early as E10 in mice ahead of the development of astrocytes (Figure 3). The blood vessels in the cerebral cortex originate from two distinct plexuses: the pial plexuses covering the brain surface and the basal vessels on the telencephalic floor [4]. The former vessels sprout into the parenchyma and grow radially from the brain surface to the deep cortical layers. The latter vessels extend tangentially from the ventral telencephalon to the cortex. After the formation of a primitive blood vessel pattern, the blood vessels actively sprout and fuse with each other to form a blood vessel network, thereby increasing the density and complexity of blood vessels over the first postnatal week [56]. This vessel remodeling concomitantly proceeds with astrocyte development, and astrocyte progenitors are highly associated with blood vessels [57]. This suggests that there is an interaction between astrocytes and blood vessels. In a study conducted by Ma et al., astrogliogenesis was genetically inhibited in the early postnatal life using GFAP-Cre;orc3-flox mice. In this animal, the density of astrocytes in the cortical gray matter progressively decreased from P3, and approximately 90% were eliminated by P7. In this situation, the density of blood vessels and their branching frequency decreased. This indicated that in angiogenesis, astrocytes are needed. Although these observations are interesting, the molecular mechanisms underlying these interactions remain largely unknown. As mentioned above, integrin β8 (Itgb8) is a key player in astrocyte-blood vessel interactions in the retina. Several genetic analyses have indicated that it is also important for the development of the cerebral cortex. In Itgb8 KO and Nestin-Cre;Itgb8-flox mice, cerebral hemorrhage and abnormal brain capillaries, including bulbous endothelial cell clusters, were observed [58,59]. However, these phenotypes were already evident at E12, indicating that they reflect a lack of Itgb8 in radial glia but not in astrocytes. Interestingly, they also observed bulbous vessels and an abnormal association of astrocytes with blood vessels at P0 [59]. Considering that nestin-Cre is also active in astrocytes [60], it is possible that some of the phenotypes might be due to the lack of astrocytic integrin β8. Integrin β8 is important for activating TGF-β in the retina. Using genetic analysis, Ma et al. demonstrated that this was also the case in the telencephalon [61]. They found that the G𝛼 co-factor Ric8a transcriptionally regulated the expression of integrin β8. They observed the presence of blood vessel defects in Nestin-Cre;ric8a-flox mice at P0, and this phenotype was reminiscent of those in Itgb8 KO mice. In fact, the expression of integrin β8 was severely decreased in the ric8a mutant. Moreover, in the mutant brains, TGF-β activity was reduced, and the signal of phospho-Smad3, an indicator of TGF-β signaling through the receptor, was concomitantly reduced in the blood vessels. In other words, Intgb8 expression under the control of ric8a in radial glia is necessary to activate TGF-β and to enhance angiogenesis in the developing telencephalon. VEGF is the main trophic factor for blood vessel development. In the cerebral cortex, radial glia or astrocytes are necessary sources of VEGF. The blood vessels in the cerebral cortex of GFAP-Cre;VEGF-flox mice were severely reduced at E17 and this defect persisted at P14 [62]. Regarding the importance of VEGF in angiogenesis of developing brains, Fantin et al. observed that in radial glia-specific VEGF gene KO mice (Nestin-Cre; Vegfa-flox) or homozygote Vegfa^120^ mutant (which lacks a domain for ECM-binding), the density of intersections and tip cells of blood vessels in the hindbrains were reduced. They also observed that microglia provide a scaffold by forming a bridge between adjacent blood vessel sprouts and supported their fusions, and macrophage depletion using Csf1^Op/Op^ mice reduced the intersections of blood vessels, but not affected tip cell density. Based on their observations that Vegfa^120^ mutation did not affect the macrophage density, they hypothesized that the sprouts required VEGF from radial glia and the anastomosis was supported by macrophage in the developing hindbrain [63].

Taken together, in the brains, VEGF derived from radial glia conducts angiogenesis during the early cortical development. In the early postnatal stages, however, radial glial cells are reduced and astrocytes are increased. There is a possibility that astrocytes promote vessel-remodeling through VEGF expression in this stage. This should be addressed in the future studies.

## 6. Blood Vessel-Guided Migration of Oligodendrocyte Progenitor Cells

Oligodendrocytes are one of the major macroglia other than astrocytes. Excellent genetic lineage tracing experiments have revealed that oligodendrocytes in the cerebral cortex are produced at different sites, depending on the developmental stage [2]. The first wave of production begins around E12.5 from Nkx2.1-expressing precursors in the medial ganglionic eminence (MGE) and anterior entopeduncular area (AEP). The second wave begins at around E15 from the Gsh2-expressing lateral ganglionic eminence (LGE) and caudal ganglionic eminence (CGE). The local production in the Emx1-expressing cortical VZ begins around birth. Direct lineage tracing using in utero electroporation of the MGE also showed a long journey of oligodendrocyte progenitor cells (OPCs) to the cerebral cortex [64]. Tsai et al. demonstrated that OPC migrate on blood vessels from the MGE to the cerebral cortex at E12 and then from the deep to superficial cortical layers at E18 in mouse embryos [65]. A similar association between OPCs and vasculature was observed in the developing human cortex at gestational week 14. They also found that OPCs interacted with blood vessels depending on the chemokine Cxcl12, which was secreted from the blood vessels, and its receptor Cxcr4, which was expressed on OPCs. The balance between migration and differentiation states was controlled by the Wnt tone. OPCs express Wnt7a and 7b, which act autonomously to activate the Wnt pathway. They keep OPCs attached to and migrated along the blood vessels depending on the Cxcl12-Cxcr4 signaling. To manipulate the Wnt tone, Tsai et al. used Olig2-Cre;Apc-flox mice, in which Wnt signaling was constitutively active and high. They observed that the expression of Cxcr4 was upregulated, and OPCs formed abnormal clusters in the blood vessels. As a scaffold, blood vessels expressed laminins α2, α4, and α5, and these ECM components supported OPC migration via the integrin β1 receptor [66]. Interestingly, it was also observed that glioma cells implanted into the neonatal rat forebrain migrated along the abluminal surface of blood vessels in a Cxcl12-Cxcr4 signaling-dependent manner [67,68,69], indicating that the infiltration of glioma in the brain was the recapitulation of the developmental process.

Undifferentiated OPCs persist throughout life in the adult CNS. Upon demyelination due to injury or pathological conditions, OPCs are activated. Subsequently, they migrate and differentiated into myelinating oligodendrocytes in the lesion [70]. Neu et al. demonstrated that adult OPCs migrated to and along blood vessels in a manner similar to the developmental processes in response to focal demyelination induced by injection of lysolecithin into the adult spinal cord or corpus callosum [71]. In this pathological situation, mice with high Wnt tone, such as Olig2-Cre;Apc-flox or PDGFRα-CreERT2;Apc-flox, formed OPC clusters on blood vessels, as observed in the developmental stages. Importantly, aberrant clusters of OPC physically displaced the astrocyte end-feet from vessels and disrupted the barrier function of the BBB. In pathological specimens of lesions from multiple sclerosis (MS) in humans, they observed perivascular clustering of OPCs, suggesting that Wnt signaling was hyperactive in MS patients. These observations imply that blood vessel can be a scaffold of migrating glia during the development and pathological conditions.

After migration, OPCs differentiate into mature oligodendrocytes and remyelinate damaged axons. Pericytes (vessel-associated PDGFRβ^+^ cells) or pericyte-like cells (vessel non-associated PEGFRβ^+^ cells) were found to play a role in this final step [72]. In response to demyelination by focal administration of lysolecithin, both cell types increased and expressed laminin α2, which in turn enhanced the differentiation of OPC into mature oligodendrocytes near the lesion. Mechanism for the differentiation of OPCs induced by laminin α2 is not clear. Integrin mediated signals or some trophic factors embedded in laminin α2 might be postulated.

## 7. Conclusions

In this paper, the complex and intimate interactions between glia and blood vessels during CNS development were overviewed. Glial and endothelial cells provide scaffolds and trophic factors to communicate with each other. As for the scaffolding between glia and endothelial cells, one of the key factors is ECM proteins, including laminins and other glycoproteins, such as FN. They bind to integrins on the plasma membrane of their counterparts. Different laminin heterotrimers specifically bind to distinct integrin heterodimers. Zoning of the ECM using these specific combinations may be essential for accomplishing complex morphogenesis. As for trophic factors secreted from glia to endothelial cells, VEGF and TGF-β are the most powerful and important factors. Interestingly, both factors are anchored in the ECM via specific interactions with ECM components, thereby forming a special niche or gradient to regulate the focal activation of the proliferation or directional growth of endothelial cells. The molecular mechanisms underlying the crosstalk between glia and endothelial cells during development have been gradually uncovered. However, especially in the developing cerebral cortex, our knowledge is still limited; hence, further studies should be conducted.

## Data Availability

Not applicable.
